# Psycho-sexual influence of sterilization on women’s quality of life: a path model

**DOI:** 10.1186/s12955-021-01733-9

**Published:** 2021-03-17

**Authors:** Samaneh Youseflu, Shahideh Jahanian Sadatmahalleh

**Affiliations:** 1 Department of Midwifery, School of Nursing and Midwifery, Zanjan University of Medical Sciences, Zanjan, Iran; 2 Department of Midwifery and Reproductive Health, Faculty of Medical Sciences, Tarbiat Modares University, Tehran, Iran

**Keywords:** Sterilization, Quality of life, Menorrhagia, Sexual function, HADS

## Abstract

**Background:**

Tubal ligation, as a permanent contraception method, have a negative and positive impact on women’s life. This study aimed to test a conceptual model considering the interrelated role of menorrhagia, body image concern, self-esteem, sexual function, anxiety and depression on quality of life (QOL) of sterilized women.

**Methods:**

The current study was conducted as a cross-sectional study on 200 sterilized women. Data were collected using a socio-demographic checklist, Short Form Health Survey (SF-12), pictorial blood loss assessment chart, female sexual function index, hospital anxiety and depression scale, body image concern inventory, and Rosenberg Self-esteem Scale. Data were analyzed using Pearson correlation coefficient and path analysis.

**Results:**

Results show that anxiety, sexual function, self-esteem, and body image dissatisfaction have a direct effect on women’s QOL. Higher level of anxiety, and body dissatisfaction directly reduce QOL. Sexual function, and menorrhagia, with an indirect effect through anxiety, reduces QOL. Higher level of anxiety with indirect effect thorough self-esteem can worsen QOL. Also, sexual function indirectly affects QOL through anxiety.

**Conclusion:**

It looks that the proposed predictors of this model are greatly important. These findings give support for a hypothetical model in which betterment in SF, body image satisfaction, self-esteem, anxiety, and menorrhagia led to a good QOL of sterilized women. Hence, in designing care for sterilized women, these factors should be considered.

## Introduction

Tubal ligation, as a permanent contraception method, have a negative and positive impact on women’s life. The reported prevalence of TL of all contraceptive methods in developing and developed countries estimated about 44% and 18%, respectively [1].

This method has several benefits for women, such as eliminated the need for long-term contraceptive pill, decreased unwanted pregnancy, endometrial and ovarian cancer [2, 3]. However, one concern of this method is risks associated with surgery, and anesthesia. On the other hand, the physical and psychological aspect of sterilization is noticeable [4, 5].

Williams and collogue described a situation that includes menorrhagia, and bleeding between menstrual cycle among sterilized women as post TL syndrome. Menstrual disorder, backache, abdominal pains, dysmenorrhea, and neurotic syndrome are more common somatic symptoms in these women [6]. The psychological consequences of this procedure are not well understood. Some women undergoing this procedure regretted it, which is the cause of distress for them [6, 7]. Various studies have shown a high prevalence of anxiety, depression, and sexual dysfunction after TL [7–9]. These factors can be effect on women’s QOL. This study aimed to test a conceptual model considering the interrelated role of anxiety, depression, body image satisfaction, sexual function, menorrhagia, and self-esteem on the QOL of sterilized women. Also, test the mediating role of anxiety and self-esteem.

## Methods

### Design and data collection

The current study was conducted as a cross-sectional study on 200 sterilized women who attended the 8 health care centers in Guilan province, Iran during 2015–2016.

Research Ethics Committees of Tarbiat Modares University of Medical Sciences (IRB # 1056668) was approved this study. After explaining the aims of the study, informed written and verbal consent was obtained from all participants. Subjects were signed that their participation was voluntary, confidential, and anonymous. The women were allocated by the convenience sampling method.

The inclusion criteria were as follows: women underwent TL at least 1 year ago, not being in the postmenopausal period, absence of the history of chronic disease includes diabetes, hypertension, thyroid and cardiovascular diseases, not using psychiatric medications, not having the history of menstrual disorders before sterilization, lake of the history of sexual abuse, no history of gynecologic surgery except caesarean section and TL, not doing breastfeeding.

Based on the literature review, the hypothesized model was assessed using Path analysis. Menorrhagia, (dependent variable); SF, anxiety, self-esteem, body image (mediators), and QOL (independent variable) were included in this model. We hypothesize that menorrhagia, SF, anxiety, self-esteem, and body image have direct effects on QOL. Also, menorrhagia, anxiety, self-esteem, and body image are predictors of SF. We also considered anxiety as a factor affecting on self-esteem, SF, and body image satisfaction.

## Measures

### Demographic and obstetric data

Socio-demographic and anthropometric characteristics including women’s age, married age, gravid, para, BMI, level of educational, history of smoking, income, job, drug and alcohol abuse, and method of delivery were collected for all subjects.

### Quality of life

The Short Form Health Survey (SF-12) containing 12 items was used to assess QOL across eight domains (includes physical function, physical role, social role, emotional role, bodily pain, general health, vitality, and mental health). The total score ranges from 0 to 100 with higher scores referring to the best condition. The psychometric properties of this questionnaire have been verified in the Iranian population [10].

### Depression and anxiety

Hospital anxiety and depression scale (HADS) questionnaire was used to evaluate the severity of anxiety and depression. This survey has 14 questions composed of two subscales that examined anxiety (7 Items) and depression (7 Items). Each item was rated on a 4-point Likert-type scale ranging from 0 to 3 (0 = never, 1 = seldom, 2 = sometimes and 3 = always) with a score range of 0–21 for both subscales. Higher scores represent greater anxiety and depression state. The validity and reliability of this questionnaire were approved among Iranian population [11].

### Sexual function

Women’s sexual function in the previous 4 weeks was measured using the female sexual function index (FSFI). This scale consists of 19 items, which assesses six main aspects of sexual functions as follows: sexual desire, arousal, lubrication, orgasm, satisfaction, and pain.

Each question was rated on a scale from 0 or 1 to 5. The sum of each domain’s scores was multiplied in its certain factor. The overall scale score ranged from 2 to 36, which higher scores meaning better sexual function. The overall scale score was obtained by adding the mean scores of all six domains, with ranged from 2 to 36. A higher scores represent better sexual function. In the Iranian population, the psychometric properties of the questionnaire have been verified [12].

### Body image concern

We used the body image concern inventory (BICI) for evaluating of the discontent and concern of the women about their appearance. This questionnaire consisted of 19 items about appearance, reassurance seeking, social concerns, and avoidance related to appearance. The answers of each item, based on the Likert spectrum, are graded from 1 (never) to 5 (always). The total score ranged from 19 to 95. Women who had a higher score are considered as a group with high body image concerns. This scale has good validity and reliability among Iranian women [13].

### Self-esteem

The Rosenberg Self-Esteem Scale was used for evaluation of women's self-esteem. This scale is a 10-items questionnaire about overall feelings of self-worth or self-acceptance.

Participants respond are ranked on 5-point Likert scale ranging from strongly agree to strongly disagree. Higher scores meaning high self-esteem. Validity and reliability of the Persian version of the questionnaire are well documented [14].

### Menorrhagia

The pictorial blood loss assessment chart (PBLAC) was used to determine the average blood loss during menstrual period. Some pads with the same brand were given to all women that participated in this study. The pictorial chart consists of diagrams that describe levels of blood on sanitary pads in three degrees: light, moderate, and heavy saturation. After swapping out the pads, according to the degree of sanitary pad stained with menstrual blood the women make a tally mark in the cell pertaining to that day of the month, and all patients used the same sanitary products. At the end of menstruation, each tick was multiplied by the corresponding coefficient (score of 1for light, 5 for moderate, and 20 for heavy staining). A total score was counted by adding together all of tally marks. PBLAC scores above 100 were defined as heavy menstrual bleeding or menorrhagia. This scale has good validity and reliability [15].

### Data analysis

Data analysis was carried out using SPSS (version 21) and LISREL software (version 8.8). Bivariate correlations were used to analyze the degree of association between the QOL, sexual function, menorrhagia, anxiety and depression, body dissatisfaction, self-esteem.

A path model was used to assess the predictive effects of independent variables on the QOL in sterilized women. Also by path analysis, we can test the cause-effect relationship between some variables. Direct, indirect, and total effects of causal relations between variables, and also values of these fit indices were computed by LISREL statistical program. For evaluation of the model fitness, RMSEA (Root mean square error of approximation), AGFI (adjusted goodness of fit index), CFI (Confirmatory Factor Analytic), and Chi-square/df were used. RMSEA values less than 0.07, Chi-square/df lower than 3, AGFI more than 0.9, and CFI more than 0.95 are indicative of a good fitting model. T-value more than + 1.96 or less than − 1.96 were considered statistically significant.

## Results

Table 1 describes the distribution of demographic and socio-economic variables of subjects. The mean age women and their partners were 35.90 ± 3.26 and 38.97 ± 3.19 years, respectively. Their mean BMI was 28.20 ± 5.09 kg/m 2, and 38.50% of participants had academic education.

**Table 1:** Demographic and anthropometric characteristics of sterilized women

Characteristic	
Age (years)*	35.90 ± 3.26
Partner age*	38.97 ± 3.19
BMI*	28.20 ± 5.09
Parity*	2.35 ± 0.56
Education**	
Lower than university	123(61.50)
University	77(38.50)
Occupation**	
Housewife	173(86.50)
Employed	27(13.50)

*BMI* body mass index

*Values are given as mean ± SD, **Values are given as number (%)

Table 2 demonstrates the Correlation (bivariate analysis) between all variables included to the path model. Results showed that QOL of women was associated with menorrhagia (r =  − 0.19, *P* < 0.01), self-esteem (r = 0.39, *P* < 0.001), body dissatisfaction (r =  − 0.27, *P* < 0.001), sexual function (r = 0.40, *P* < 0.001), and anxiety and depression (r =  − 0.62, *P* < 0.001).

**Table 2:** Correlations between anxiety, self-steem, body image satisfaction, sexual function, age, BMI, and quality of life of infertile women

	1	2	3	4	5
1. Quality of life	–	–	–	–	–
2. Menorrhagia	− 0.19^**^	–	–	–	–
3. Self-esteem	0.39^***^	− 0.12*	–	–	–
4. Body dissatisfaction	− 0.27***	− 0.02	− 0.21**	–	–
5. Sexual function	0.40***	0.12	0.22**	− 0.13	–
6. Anxiety	− 0.62***	0.15*	− 0.32***	0.21^**^	− 0.38^***^

Values are given as Pearson coefficient (*P* value) using Pearson correlation test

^*^P < 0.05; **P < 0.01; ***P < 0.001

The overall goodness-of-fit statistics showed that the conceptual model of the study was excellent (*P* value = 0.03; chi2 = 15.77; DF = 5; chi2/*df* = 3.15; RMSEA = 0.07; CFI = 0.98; GFI = 0.95) (Table 3).

**Table 3:** The goodness of fit indices for the models

	CFI*	GFI**	RMSEA***	Chi-square	df	Chi-square/df****	*P* value
Path N = 200	0.98	0.95	0.07	15.77	5	3.15	0.03

*CFI: comparative fit index, **GFI: goodness fit index, ***RMSEA: root mean square error of approximation, ****Chi-square/*df*: chi-square to the degree of freedom index

**Fig. 1:**
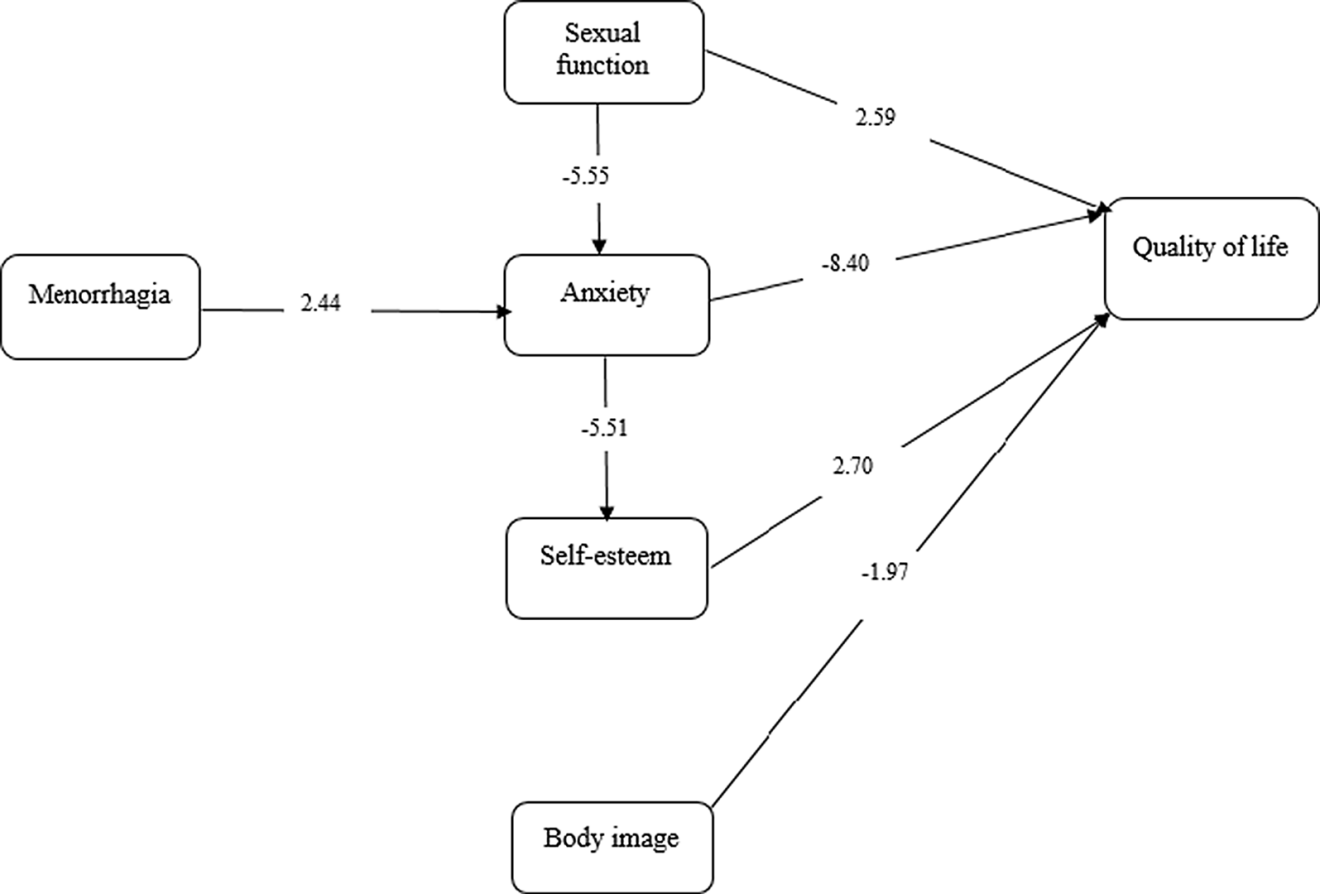
Path diagram for the predictors of QOL in sterilized women. *Values are reported based on T-value using LISREL software

Table 4, and Fig. 1 shows the direct, indirect, and total effects of variables on women's QOL after TL. Results show that anxiety (β =  − 0.51), sexual function (β = 0.15) self-esteem (β = 0.15), and body image dissatisfaction (β =  − 0.10) have a direct effect on women’s QOL. Higher level of anxiety, body dissatisfaction with direct effect can be reduce QOL. Anxiety (β =  − 0.05), sexual function (β = 0.20), and menorrhagia (β =  − 0.09) with indirect effect impress QOL.

**Table 4:** Direct, indirect, and total effect of some variables on QOL of sterilized women

	Direct effect	Indirect effect	Total effect
Menorrhagia	− 0.02	− 0.09	− 0.11
Sexual function	0.14	0.20	0.34
Anxiety and depression	− 0.50	− 0.05	− 0.55
Self-esteem	0.15	–	0.15
Body image dissatisfaction	− 0.10	–	− 0.10

Among variables anxiety and depression have more direct effect (β =  − 0.50) on QOL. The most indirect effect was related to sexual function (β = 0.20). Women who had higher score in menorrhagia (β = 0.16), and low sexual function (β = − 0.36) were more anxious. Higher level of anxiety with indirect effect thorough self-esteem can worsen QOL (β = − 0.05).

## Discussion

Recently, several studies were conducted to explore the factors affecting the QOL of sterilized women. There is controversy regarding the strength of the relationship between psychosexual variables and women's QOL due to uncontrolled interrelationships with various effects of modifiers, mediators or confounding variables on QOL in sterilized women.

Moreover, it seems that usual statistical techniques, due to the high colinearity level between variables, cannot accurately estimate the relationship strengths and interrelationships of psychosexual variables with impairment of QOL.

Utilizing a developed conceptual model (path diagram) for both exploratory and confirmatory aims by providing testing of the associations between the basic concepts of the theory has been proposed to reduce the limitations of the traditional statistical methodologies.

The results of this study indicated that anxiety and depression, self-esteem, menorrhagia, body image satisfaction, and sexual function were significant along the pathway of predictors of QOL in sterilized women.

There are conflicting results on the influence of sterilization on women’s QOL [16, 17]. Alyahya et al. study revealed that women who had undergone permanent sterilization had lower QOL scores in all of the four domains. Also, experience side effects and vaginal bleeding in these women are related to QOL impairment [18]. In contrast, the results of Li et al. [16] study showed that female sterilization have a positive impact on women’s QOL.

In our study sexual function is one of the predictors of QOL. Moreover, sexual function with indirect effect thorough reduce anxiety improve QOL. Various study shows controversial results about the role of sterilization on sexual function [9, 16, 19]. The results of one cohort study on 4576 sterilized women revealed that over 80% of women did not report any consistent changes in sexual life after sterilization, nevertheless, positive effects were more common than negative in women with consistent change [20]. On the other hand, Jahanian et al. study demonstrates that negative changes in sexual function after sterilization is one important factor in the impairment of QOL [8]. It seems that physical discomfort and pain, as well as severe bleeding after TL, leads to impairment of women's sexual function [5, 6]. Adverse effects of sexual dysfunction on women’s sense of wholeness, confidence, social relations, and marital status are noticeable [8].

Menorrhagia was a more common symptom in sterilized women. Our result indicated that menorrhagia indirectly affects women’s QOL through anxiety. A negative impact of menorrhagia on the QOL has been previously discussed by other authors [21, 22].

We also observe women with menorrhagia had more psychological impairment (i.e., anxiety and depression).

One of the main serious stressors in women's lives is reproductive problems. Sterilization with impeding the woman’s identity achievement as a mother and the fulfillment of desired life goals is associated with increased distress for women. Regret after tubal sterilization is a main factor in developing psychological distress [4]. The results surrounding the influence of sterilization on anxiety levels are controversial [4, 23, 24]. Kelekçi et al. [4] study show a higher prevalence of anxiety and depression in women after sterilization. On the contrary, Cooper et al. found that sterilization can reduce the risk of psychological problems [24].

Our results have revealed a higher level of anxiety and depression with a direct effect reduce women’s QOL. Anxiety also with indirect effect thorough low self-esteem affects QOL. Sterilized women with low self-esteem had a lower level of QOL.

Body image is one of the important concepts of psychology that includes “affective, cognitive, behavioral, and perceptual features” [25]. We observe that a higher level of body image dissatisfaction with direct effect has a negative effect on QOL. For women, the childbearing function was considered as an integral part of the body image. It seems that TL as a type of infertility changes women’s body image. The existence of body image disturbance includes loss of femininity, less sexually appealing, dissatisfaction with appearance, poorer wellness behaviors may have negative consequences on the QOL and psychosocial health [26, 27].

Whereas various studies only have examined the direct effect of psycho-sexual consequences of TL on women's life. The current study was the first to consider these factors in tandem and was estimated the direct, indirect, and total effects of each of the variables on QOL. The combination of the assessed domains is the main strength of the current study.

Using a developed conceptual model, and the use of validated questionnaires (eg, PBLAC, FSFI, SF-12, BICI, Rosenberg Self-Esteem Scale, and HADS) are other strengths of this study.

Despite its strengths, the present study contains several limitations. First, participants were selected using a convenience sampling method. Second, we did not evaluate the influence of other variables (such as marital satisfaction, type of personality, intrapersonal relationship with partner, partner violence, sexual knowledge, etc.) that can affect women's QOL. It has been proposed, that future studies consider these issues.

## Conclusion

Regarding the psycho-sexual influence of TL on women’s QOL, a comprehensive consultation about the negative consequence of sterilization before TL, and psychotherapeutic and psychosexual help after sterilization should be offered.

## Data Availability

The data sets used and analyzed during the current study are available from the corresponding author on reasonable request.

## Abbreviations

QOL: Quality of life
TL: Tubal ligation
BICI: Body image concern inventory
HADS: Hospital anxiety and depression scale
PBLAC: Pictorial blood loss assessment chart
